# Identification of a novel metabolism-related gene signature associated with the survival of bladder cancer

**DOI:** 10.1186/s12885-021-09006-w

**Published:** 2021-11-24

**Authors:** Xiaotao Li, Shi Fu, Yinglong Huang, Ting Luan, Haifeng Wang, Jiansong Wang

**Affiliations:** 1 Department of Urology, The Second Affiliated Hospital of Kunming Medical University, No. 347, Dianmian street, Wuhua District, Kunming, 650101 Yunnan People’s Republic of China; 2 Urological disease clinical medical center of yunnan province, The Second Affiliated Hospital of Kunming Medical University, No. 347, Dianmian street, Wuhua District, Kunming, 650101 Yunnan People’s Republic of China; 3 Scientific and Technological Innovation Team of Basic and Clinical Research of Bladder Cancer in Yunnan Universities, The Second Affiliated Hospital of Kunming Medical University, No. 347, Dianmian street, Wuhua District, Kunming, 650101 Yunnan People’s Republic of China

**Keywords:** Bladder cancer, Metabolism-related gene, TCGA, GEO, Prognosis

## Abstract

**Background:**

Bladder cancer (BC) is one of the most common malignancies and has a relatively poor outcome worldwide. In this study, we attempted to construct a novel metabolism-related gene (MRG) signature for predicting the survival probability of BC patients.

**Methods:**

First, differentially expressed MRGs between BC and normal samples were identified and used to construct a protein-protein interaction (PPI) network and perform mutation analysis. Next, univariate Cox regression analysis was utilized to select prognostic genes, and multivariate Cox regression analysis was applied to establish an MRG signature for predicting the survival probability of BC patients. Moreover, Kaplan-Meier (KM) survival analysis and receiver operating characteristic (ROC) analysis were performed to evaluate the predictive capability of the MRG signature. Finally, a nomogram based on the MRG signature was established to better predict the survival of BC.

**Results:**

In the present study, 27 differentially expressed MRGs were identified, most of which presented mutations in BC patients, and LRP1 showed the highest mutation rate. Next, an MRG signature, including MAOB, FASN and LRP1, was established by using univariate and multivariate Cox regression analysis. Furthermore, survival analysis indicated that BC patients in the high-risk group had a dramatically lower survival probability than those in the low-risk group. Finally, Cox regression analysis showed that the risk score was an independent prognostic factor, and a nomogram integrating age, pathological tumor stage and risk score was established and presented good predictive ability.

**Conclusion:**

We successfully constructed a novel MRG signature to predict the prognosis of BC patients, which might contribute to the clinical treatment of BC.

**Supplementary Information:**

The online version contains supplementary material available at 10.1186/s12885-021-09006-w.

## Background

Bladder cancer (BC), a malignancy of the urinary tract, is the 10th most prevalent tumor worldwide, with 549,000 newly diagnosed cases and 200,000 deaths estimated in 2018 [1]. It was reported that urothelial carcinoma accounts for 95% of BC, and most BC patients are nonmuscle-invasive, mainly treated through local treatment or surveillance [2, 3]. However, the clinical treatments of the remaining patients without metastasis primarily rely on surgical resection or a combination of local resection, radiation, and chemotherapy [3]. Although the main therapy for BC patients with unresectable or metastatic disease is platinum-based combination chemotherapies, the survival remains poor, with a median overall survival (OS) of approximately 14 months [4, 5]. On the other hand, even though immune checkpoint blockade has emerged as a novel treatment to extend the OS of some patients, immune checkpoint blockade regrettably does not work in most patients [6]. Therefore, it is essential to recognize novel biomarkers to predict the prognosis and guide the treatment of BC.

Metabolism plays a crucial role in maintaining all biological activities and regulating cell growth and proliferation [7, 8], which has attracted the attention of many researchers in recent years. Cancer cells have unique metabolic characteristics that can satisfy the need for proliferation compared to normal cells [9]. A well-known example of this is the Warburg effect, a unique reprogramming form of glucose metabolism, which can promote the occurrence and development of tumors [10, 11]. On the other hand, the energy demand for meeting the survival of cancer cells in the nutrient-deprived tumor microenvironment relies on metabolic alterations [12]. Recent studies have found that fatty acid metabolism is related to the development of BC [13–15]. Moreover, increasing evidence has revealed that glucose metabolism is also associated with the occurrence and progression of tumors [16, 17]. For example, aberrant expression of miRNA-21 might participate in regulation of the glycolytic phenotype in BC cells [17]. FOXJ1 also has a role in the glycolytic phenotype of BC [18]. Furthermore, the epigenetic perturbation of SAT1 and ASS1 may be involved in the chemotherapy and personalized therapy of BC by regulating its amino acid metabolism [19]. Hence, metabolism plays a vital role in the occurrence and development of BC, and research focusing on metabolism-related genes (MRGs) may contribute to further understanding the role of metabolism in BC and identifying novel therapeutic targets.

Currently, more and more studies have explored the association between MRGs and the prognosis of cancers. For instance, Wen et al. established a model to predict the prognosis of gastric cancer patients based on MRGs [20]. Moreover, a risk model with good performance in the prognostic prediction of hepatocellular carcinoma patients was built based on energy metabolism genes [21]. Wu et al. also found that lipid metabolism-related genes could be used as predictors for the survival of diffuse gliomas [22]. Hence, the present study aimed to establish an MRG signature for predicting the survival of BC based on a series of bioinformatics analyses using the Gene Expression Omnibus (GEO) and The Cancer Genome Atlas (TCGA) databases. Moreover, we further validated the mRNA expression level of genes in the MRG signature through real-time PCR. In brief, the establishment of an MRG signature provided a novel and independent predictor for BC survival and might be conducive to the clinical treatment of BC patients.

## Methods

### Data collection and processing

The GSE13507 dataset was downloaded from the GEO database, which included 9 healthy controls and 165 patients with BC, and the GSE31684 dataset, which included 93 BC patients with survival information, was extracted from the GEO database as an independent validation set. Moreover, gene expression, somatic mutations and clinical data of 414 BC samples were obtained from the TCGA database, and gene expression data of 19 normal samples were obtained as controls from the TCGA database. Next, we downloaded the gene set (h.all.v7.2.entrez.gmt) from the GSEA website (https://www.gsea-msigdb.org/gsea/index.jsp) to screen MRGs by enrichment analysis with the ‘clusterProfiler’ R package.

### Identification of metabolism-related DEGs in BC

The ‘DEseq2’ R package was selected to identify the differentially expressed genes (DEGs) between normal and BC samples in the TCGA and GEO databases [23]. A *P* value < 0.05 was regarded as the cutoff criterion. Moreover, two volcano plots were plotted using the ‘ggplot2’ R package to visualize DEGs in the TCGA and GEO databases [24]. Finally, the metabolism-related DEGs were identified by overlapping the MRGs, DEGs in TCGA and DEGs in GEO using the ‘VennDiagram’ R package [25].

### GO functional annotation and KEGG pathway enrichment analysis

Gene Ontology (GO) functional annotation is an important method to explore the biological process (BP), molecular function (MF), and cellular component (CC) of genes. Moreover, Kyoto Encyclopedia of Genes and Genomes (KEGG) pathway enrichment analysis is a common way to identify gene-related signaling pathways. Therefore, the ‘clusterProfiler’ R package was utilized to conduct GO functional annotation and KEGG pathway enrichment analysis for the metabolism-related DEGs [26], and a *P value* < 0.05 was considered to be significantly enriched.

### PPI network analysis

To further explore the interactions of the metabolism-related DEGs at the protein level, a protein-protein interaction (PPI) network was built through the Search Tool for the Retrieval of Interacting Genes (STRING, https://string-db.org/) website. In addition, Cytoscape was used to visualize the PPI network [27].

### Landscape of gene mutations

To further investigate the role of metabolism-related DEGs in BC, the ‘maftools’ R package was used to analyze the mutation frequency and mutation type of BC patients from the TCGA database and to draw a waterfall plot showing the landscape of gene mutations for metabolism-related DEGs [28].

### Construction and validation of the prognostic MRG signature

To establish and validate the prognostic MRG signature, 383 BC patients (31 patients were removed for subsequent analysis because of a lack of survival information) were randomly divided into a training set and a testing set based on a ratio of 7:3. First, univariate Cox regression analysis was performed using the ‘survival’ R package to screen the prognosis-related MRGs from the metabolism-related DEGs in the training set, with the a cutoff value of *P* < 0.05. Then, prognosis-related MRGs were submitted to multivariate Cox regression analysis to construct an optimal prognostic MRG signature in the training set by the ‘survival’ R package. Forest plots were generated to show the results of univariate and multivariate Cox regression analysis by using the ‘forestplot’ R package. Subsequently, an MRG signature was constructed based on the expression levels and Cox coefficients of the MRGs obtained by multivariate Cox regression analysis. Namely, the formula of the risk score for the MRG signature was defined as follows: Risk score = (exp_gene 1_ × Coe_gene 1_) + (exp_gene 2_ × Coe_gene 2_) + … + (exp_gene n_ × Coe_gene n_). Thus, BC patients in the training set, testing set and validation set were stratified into the high-risk and low-risk groups based on the median risk score value of the MRG signature. Moreover, the Kaplan-Meier (KM) survival curves were drawn by the ‘survminer’ R package to reveal the OS for patients in the high-risk and low-risk groups, and the log-rank test was used to analyze significant differences in OS. Receiver operating characteristic (ROC) curves were plotted to assess the prediction accuracy of the MRG signature and the area under the curve (AUC) for 1-year, 3-year and 5-year OS was calculated through the ‘survivalROC’ R package [29].

### Association between the MRG signature and clinicopathological features

The association between the MRG signature and clinicopathological features, including gender, age, pathological tumor stage, pathological T stage, pathological M stage, pathological N stage, was calculated by t test in the training set, and *P* < 0.05 was considered statistically significant. In addition, a cluster heatmap was drawn to show the distribution trends of gender, age, pathological tumor stage, pathological T stage, pathological M stage, and pathological N stage between the low-risk and high-risk groups in the training set.

### Construction of predictive nomogram

The MRG signature and clinicopathological features were used to identify independent prognostic factors with univariate and multivariate Cox regression analyses in the training set, and the results of univariate and multivariate Cox regression analyses are by forest plots. Next, a nomogram was constructed by independent prognostic factors through the ‘rms’ R package [30]. Moreover, calibration plot was plotted to assess the predictive ability of the nomogram.

### Quantitative real time PCR validation

To further analyze the roles of genes in the MRG signature, we first examined the expression levels of genes in the MRG signature in the TCGA and GEO databases. Next, we collected 10 cancer tissues and 10 pericarcinomatous tissues from BC patients in The Second Affiliated Hospital of Kunming Medical University. Informed consent was obtained from all participating individuals. The study was approved by the Ethics Committee at The Second Affiliated Hospital of Kunming Medical University.

Total RNA from the 20 samples was extracted by TRIzol-A+ Reagent (TIANGEN) based on the manufacturer’s guidance. Then, the RNAs were reverse-transcribed into complementary DNA (cDNA) using the FastQuant RT Kit (TIANGEN) according to the manufacturer’s procedure. Real-time PCR was performed by upeReal PerMix Plus (SYBRGreen) (TIANGEN) and the Applied Biosystems 7500 Real-time PCR System (Applied Biosystems, Inc., Carlsbad, CA, United States). Through the 2-^ΔΔCt^ method, the relative expression of genes was calculated. GAPDH was used as an internal references. Primer sequences and annealing temperatures are summarized in Table S1.

### Statistical analysis

All statistical analyses in this study were performed using R software. The differences between different groups were compared by the t test and log-rank test. A *P value* < 0.05 was considered statistically significant.

## Results

### Identification of metabolism-related DEGs in BC

**Fig. 1:**
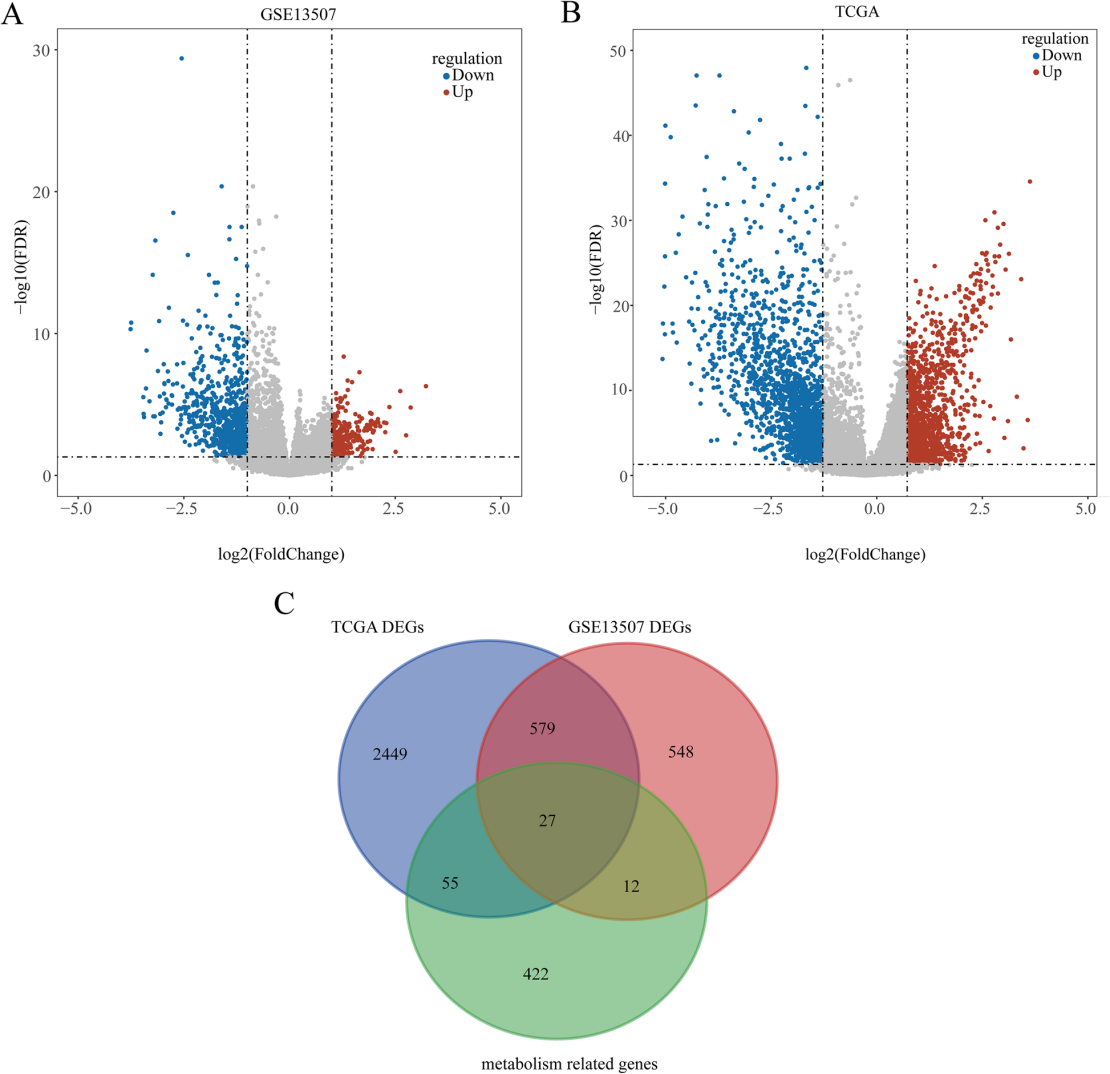
Identification of Metabolism-related DEGs. **A B** The volcano plots of DEGs in normal samples compared to BC samples in GSE13507 and TCGA database (**B**). **C** The venn diagram of MRGs, DEGs in GSE13507 and DEGs in TCGA database

We performed functional annotation of the h.all.v7.2.entrez.gmt gene set and obtained 516 MRGs (Table S2). Moreover, under the cutoff of *P* < 0.05, 1166 DEGs, including 288 upregulated genes and 878 downregulated genes, were identified between normal and BC samples in the GEO database (Fig. 1A, Table S3), and 3110 DEGs, including 1306 upregulated genes and 1804 downregulated genes were identified between BC patients and normal samples collected from the TCGA database (Fig. 1B, Table S4). Finally, 27 metabolism-related DEGs were identified, including 19 downregulated and 8 upregulated genes, by overlapping the genes among MRGs, DEGs in GSE13507 and DEGs in the TCGA database (Fig. 1C, Table S5).

### GO functional annotation and KEGG pathway enrichment analysis of metabolism-related DEGs

**Fig. 2:**
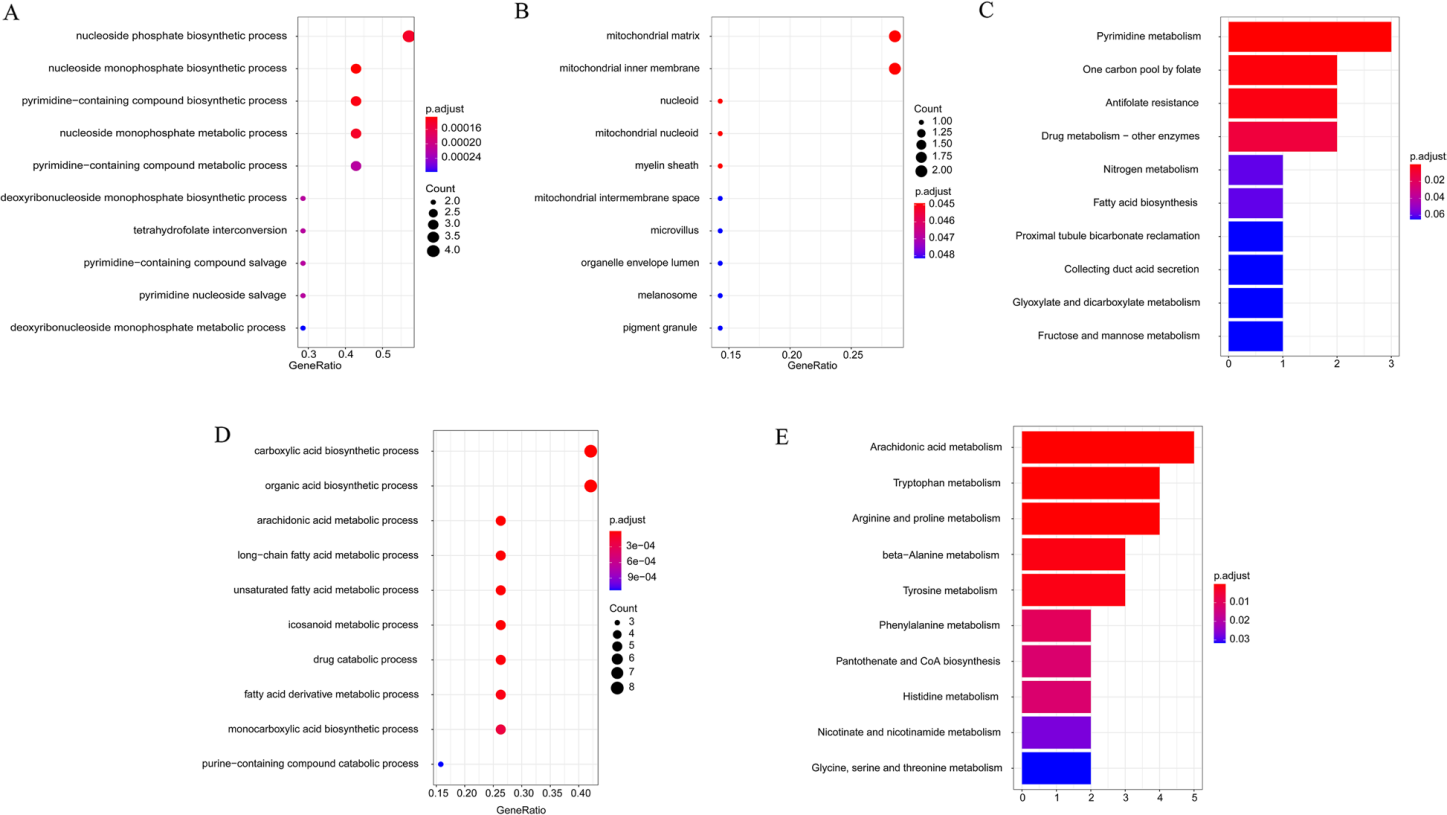
The results of GO Functional Annotation and KEGG Pathway Enrichment Analysis. **A** The enriched biological processes by metabolism-related DEGs. **B** The enriched cellular components by metabolism-related DEGs. **C** The enriched molecular functions by metabolism-related DEGs. **D** The enriched KEGG pathways by metabolism-related DEGs

To better understand the biological function of 27 metabolism-related DEGs, we conducted GO function and KEGG enrichment analyses were conducted. The results of BP for GO analysis showed that 8 upregulated metabolism-related DEGs were primarily associated with biosynthesis and metabolism, for example, nucleoside phosphate biosynthetic process, nucleoside monophosphate biosynthetic process, pyrimidine-containing compound biosynthetic process, nucleoside monophosphate metabolic process, and pyrimidine-containing compound metabolic process (Fig. 2A). For CC analysis, the 8 upregulated metabolism-related DEGs were significantly involved in the mitochondrial matrix, mitochondrial inner membrane, and nucleoid (Fig. 2B). In addition, for MF, the 8 upregulated metabolism-related DEGs were not significantly enriched. In particular, KEGG analysis showed that the 8 upregulated metabolism-related DEGs were mainly enriched in metabolism-related pathways, such as pyrimidine metabolism, drug metabolism, nitrogen metabolism, and glyoxylate and dicarboxylate metabolism (Fig. 2C). Similarly, the results of BP for GO analysis showed that the 19 downregulated metabolism-related DEGs were mainly related to biosynthesis and metabolism, such as carboxylic acid biosynthetic process, organic acid biosynthetic process, arachidonic acid metabolic process and long-chain fatty acid metabolic process (Fig. 2D). For CC and MF analysis, the 19 downregulated metabolism-related DEGs were not significantly enriched. Moreover, KEGG analysis showed that the 19 downregulated metabolism-related DEGs were mainly enriched in metabolism-related pathways, such as arachidonic acid metabolism, tryptophan metabolism, arginine and proline metabolism, beta-alanine metabolism, and tyrosine metabolism (Fig. 2E). Thus, these results further suggested that 27 metabolism-related DEGs were mainly related to metabolism-related biological processes and signaling pathways.

### PPI network

**Fig. 3:**
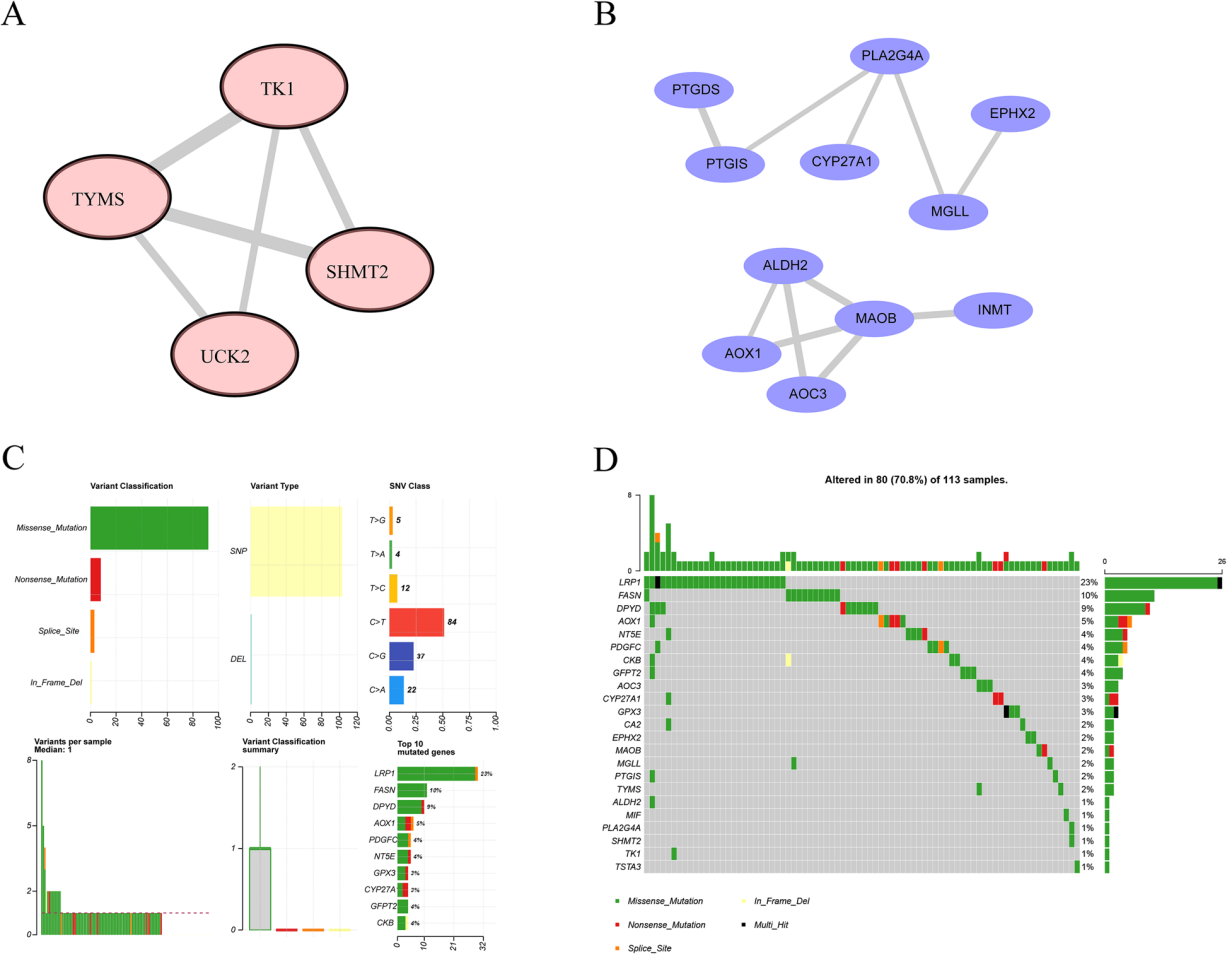
PPI network genetic variation for metabolism-related DEGs. **A** The PPI network of 27 metabolism-related DEGs. **B** The bar diagrams showed the interactions of each gene and other genes. **C** The mutation frequency of 23 metabolism-related DEGs in 414 BC samples from the TCGA database

To further observe the interactions among 27 metabolism-related DEGs, we constructed an up-PPI network and a down-PPI network. As shown in Fig. 3A, upregulated TK1, TYMS, UCK2 and SHMT2 directly interacted with each other. Moreover, downregulated PTGDS, TTGIS, PLA2G4A, CYP27A1, MGLL and EPHX2 directly or indirectly interacted with each other, and ALDH2, AOX1, AOC3, MAOB, and INMT directly or indirectly interacted with each other (Fig. 3B).

### Landscape of genetic variation for metabolism-related DEGs

To further investigate the roles of 27 metabolism-related DEGs in the BC, we further analyzed the landscape of somatic mutations for 27 metabolism-related DEGs using somatic mutation data of 414 BC samples from the TCGA database. Notably, among 27 metabolism-related DEGs, most genes experienced mutations in BC patients, and the LRP1 showed the highest mutation frequency (Fig. 3C). Notably, missense was the primary mutation type (Fig. 3C and D). Moreover, the top 10 mutated genes were LRP1, FASN, DPYD, AOX1, PDGFC, NT5E, GPX3, CYP27A1, GFPT2, and CKB (Fig. 3D). In addition, survival differences were compared between mutated and nonmutated samples of each gene. Interestingly, we found that GPX3 and SHMT2 mutations were related to BC prognosis (*P value* < 0.05, Table S6). These results further revealed that metabolism-related genes might play key roles in BC.

### Construction and validation of the prognostic MRG signature

**Fig. 4:**
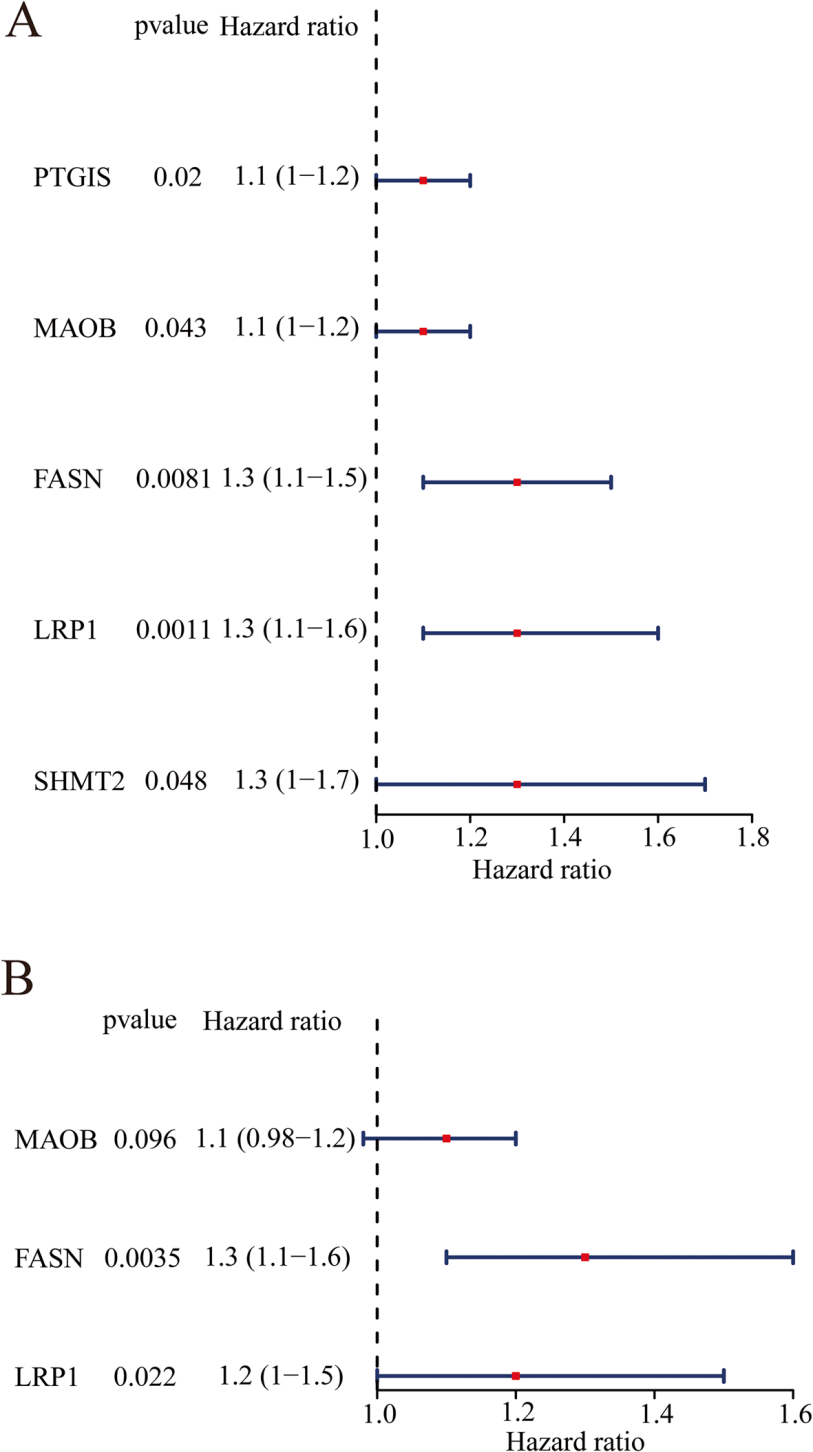
Identification of prognostic metabolism-related DEGs. **A** Univariate Cox regression analysis identified 5 prognostic metabolism-related DEGs. **B** Multivariate Cox regression analysis reserved 3 prognostic metabolism-related DEGs for establishing the prognostic MRG signature

**Fig. 5:**
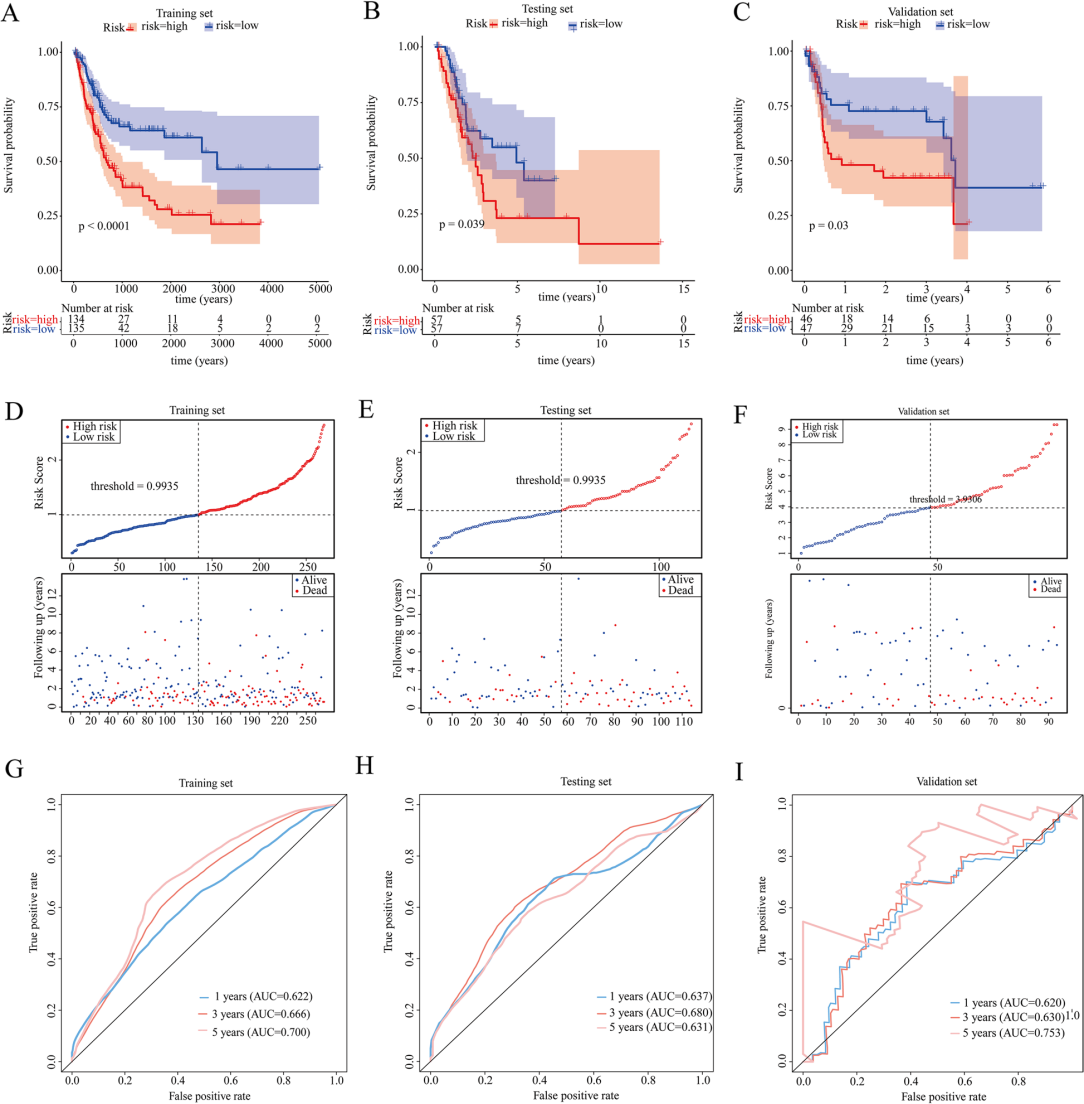
Assessing the efficiencies of the prognostic MRG signature in the training set and validation set. **A B C** The Kaplan-Meier survival curves of the training set (**A**), the testing set (**B**) and the validation set (**C**). **D E F** The distribution of risk scores and the survival status of patients in the training set (**D**), the testing set (**E**) and the validation set (**F**), and each dot represents a BC patient. **G H I** ROC curves of the training set (**G**), the testing set (**H**) and the validation set (**I**) showed the performance for predicting the 1-year, 3-year and 5-year OS

The 383 BC patients were randomly divided into a training set and validation set at a cutoff of 7:3. First, univariate Cox regression analysis identified 5 prognostic MRGs, including PTGIS, MAOB, FASN, LRP1 and SHMT2 (*P value* < 0.05, Fig. 4A), in the training set. Next, 3 genes, MAOB, FASN and LRP1, were reserved to establish a prognostic MRG signature based on the multivariate Cox regression analysis in the training set (Fig. 4B). MAOB, monoamine oxidase B, an enzyme located on the outer membranes of mitochondria, is responsible for catalyzing monoamine oxidation. FASN, fatty acid synthase, is mainly involved in fatty acid synthesis. LRP1, LDL Receptor Related Protein 1, is associated with several cellular processes, including intracellular signaling, lipid homeostasis, and clearance of apoptotic cells. Notably, all 3 genes were risk factors for BC survival with HR > 1 (Fig. 4B), indicating that higher expression of MAOB, FASN and LRP1 was related to poorer prognosis. Thus, we further plotted the KM survival curves of MAOB, FASN and LRP1 in the training, testing and validation sets and found that patients in the high expression group showed a worse prognosis than patients in the low expression group (Fig. S1). Next, the formula used to calculate the risk score was as follows: Risk score = (0.088 × MAOB expression level) + (0.286 × FASN expression level) + (0.214 × LRP1 expression level). The patients in the training set were stratified into a high-risk group and a low-risk group based on the median risk score value. As shown in Fig. 5A, patients in the high risk group showed significantly poorer OS than those in the low-risk group. Consistently, the patients in the high-risk group appeared to have a higher mortality than patients in the low-risk group (Fig. 5D). Moreover, the ROC curve suggested that the MRG signature could accurately predict the 1-year, 3-year and 5-year OS, and the AUC values for predicting the 1-year, 3-year and 5-year OS were 0.622, 0.666, and 0.700, respectively (Fig. 5G). Furthermore, based on the formula mentioned above, the patients in the testing set and validation set were stratified into a high-risk group and a low-risk group according to the median risk score value, respectively. Consistent with the results of the training set, the patients in the high-risk group also presented significantly worse OS than those in the low-risk group in testing set and validation set (Fig. 5B, C, E and F). Similarly, ROC curves of the testing set and validation set also showed better accuracy for predicting the 1-, 3- and 5-year survival of BC patients (Fig. 5H and I), and the AUC values in the testing set were 0.637 at 1 year, 0.680 at 3 years and 0.631 at 5 years (Fig. 5H). Meanwhile, those in the validation set were 0.620, 0.630 and 0.753, respectively (Fig. 5I). Therefore, these results indicated that the MRG signature presented good performance for predicting the OS of BC patients.

### Correlation between the MRG signature and clinicopathological characteristics

To explore the role of the MRG signature in the progression of BC, the association between the MRG signature and clinicopathological characteristics was investigated in the training set. As shown in Table 1, the patients in the high-risk group were inclined to include more patients older than 60. Moreover, the patients in the high-risk group appeared to contain more high-grade BC (including pathological tumor stage 3 and 4) (Table 1). Thus, the gene signature may be associated with the progression of BC, and the expression levels of risk genes might be influenced by the age of patients.

**Table 1:** Clinicopathological characteristics of patients in high- and low-risk group in the training set

Characteristics	Number	Risk score		*P*-value
		Low	High	
Total cases	219	109	110	
Gender				
female	44	18	26	0.2520
male	175	91	84	
Age				
> =60	174	80	94	**0.0412**
< 60	45	29	16	
Pathological tumor stage				
1	2	1	1	**0.0165**
2	60	40	20	
3	79	37	42	
4	78	31	47	
T stage				
T1	2	1	1	0.0629
T2	69	43	26	
T3	117	49	68	
T4	31	16	15	
M stage				
M1	105	46	59	0.2300
M2	108	60	48	
M3	6	3	3	
N stage				
Nx	15	6	9	0.1090
N0	129	74	55	
N1	30	12	18	
N2	41	16	25	
N3	4	1	3	

### Construction of a nomogram for predicting the OS of BC

**Fig. 6:**
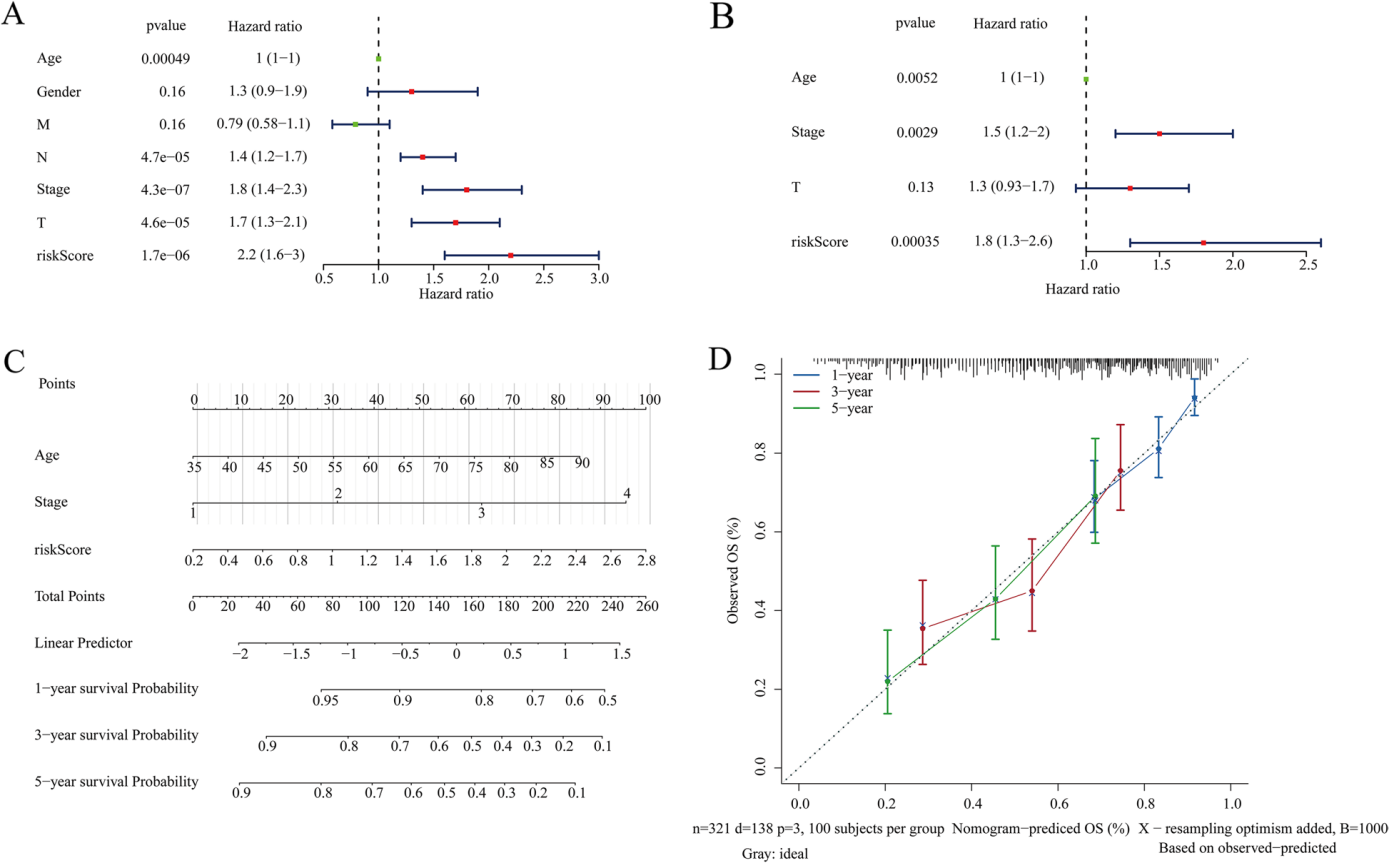
Construction of a nomogram for better predicting the 1-year, 3-year and 5-year OS of patients in the training set. **A B** Univariate (**A**) and multivariate (**B**) Cox regression analyses identified independent prognostic factors in training set. **C** Nomogram based on the age, pathological tumor stage and risk score was established in the training set. **D** The calibration curve showed the predictive efficiency of nomogram in the training set

To better use the gene signature, a nomogram combining the gene signature and clinical features was established to predict the OS of BC patients. First, univariate and multivariate Cox regression analyses were performed to screen independent prognostic factors in the training set. The results of univariate Cox regression analysis suggested that age, gender, pathological tumor stage, pathological T stage, pathological M stage, pathological N stage and risk score were responsible for the OS of BC (*P value* < 0.05, Fig. 6A). Next, multivariate Cox regression analysis indicated that age, pathological tumor stage, and risk score could be used to establish a nomogram via the quantitative scoring method (*P value* < 0.05, Fig. 6B and C). Furthermore, the calibration curve suggested that the nomogram showed good agreement between the predicted OS and observed OS (Fig. 6D). Thus, the nomogram had good accuracy for predicting the 1-year, 3-year and 5-year survival rates of BC patients.

### Quantitative real time PCR validation

**Fig. 7:**
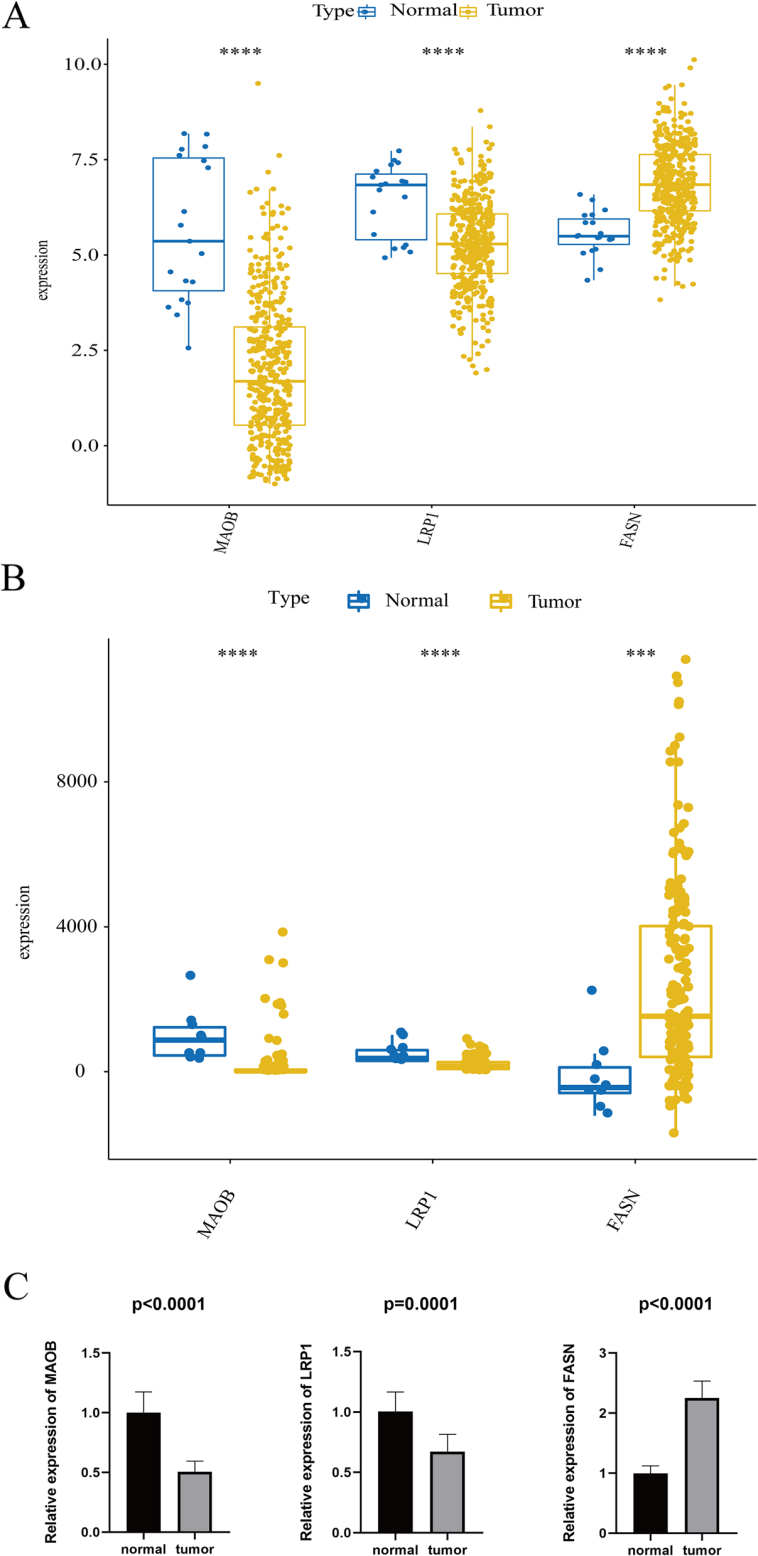
The expression levels of MAOB, FASN and LRP1. **A** TCGA database. **B** GSE13507. **C** Clinical samples

To further investigate the expression levels of MAOB, LRP1 and FASN, we performed quantitative real-time PCR validation. Notably, the expression levels of both MAOB and LRP1 were downregulated in tumor samples compared with normal samples in the TCGA and GEO databases (Fig. 7A and B), but the expression of FASN was upregulated. Consistent with the TCGA and GEO results, we also found that the expression levels of MAOB and LRP1 were downregulated in cancer tissues compared with paracarcinoma tissues, and the expression of FASN was upregulated (Fig. 7C). Thus, MAOB, LRP1 and FASN might be good biomarkers for BC.

## Discussion

BC is a common malignancy of the urinary tract worldwide and has an approximately three times higher morbidity in men than in women [31, 32]. Although most patients (> 70%) have nonmuscle-invasive BC at the initial diagnosis, the high recurrence rate greatly reduces the prognosis of patients and 10–15% of them will eventually progress to the muscle-invasive stage [33–35]. Currently, with the development of surgery, chemotherapy and immunological therapy, the clinical management of BC has undergone major improvement [36, 37]. Nevertheless, the prognosis of BC patients remains poor, and the efficacy of immunological therapy still needs improvement because it only benefits a small proportion of patients [6, 38]. Therefore, screening novel biomarkers to predict the prognosis of BC and treatment remains urgent and challenging. Increasing evidence has revealed that metabolic imbalance can influence the growth, proliferation, angiogenesis, and invasion of cancer cells [39–41]. Although recent research has revealed that all types of metabolic pathways, including glucose, lipids, amino acids, nucleotides and other pathways, may act as potential prognostic markers of BC [42], few studies have focused on the regulation of metabolism at the molecular level. Hence, the present study aimed to systematically investigate the role of MRGs in the occurrence and progression of BC and screen biomarkers for predicting the OS of BC.

In the present study, we first identified 27 differentially expressed MRGs by overlapping the MRGs, DEGs in TCGA, and DEGs in GEO (Fig. 1C). Next, we investigated the biological functions of these 27 differentially expressed MRGs and found that they were mainly associated with metabolism (Fig. 2). Thus, we speculated that these genes might play key roles in BC by activating or inhibiting metabolism-related pathways, and ultimately affecting the substances and energy which are necessary for tumor cells growth and reproduction of tumor cells. Moreover, we further explored their interactions and genetic changes and found that several genes could interact with each other (Fig. 3A and B), and most genes experienced mutations in BC patients (Fig. 3C and D). Moreover, an MRG signature with good performance, including MAOB, FASN and LRP1, was established to predict the OS of BC patients. Notably, the gene signature was associated with age and tumor stage (Table 1). Finally, we constructed a nomogram to better use the gene signature and further validated the expression levels of MAOB, FASN and LRP1. Interestingly, the results of quantitative real-time PCR were consistent with the results of TCGA and GEO. Namely, the expression levels of MAOB and LRP1 were downregulated in cancer tissues compared with paracarcinoma tissues, and the expression of FASN was upregulated (Fig. 7).

MAOB, encoding an enzyme that can generate hydrogen peroxide by oxidative reaction, is mainly associated with neurotransmitter metabolism-related biological processes [43]. In the present study, we found that higher expression of MAOB was associated with worse survival in BC patients (Fig. 4B and Fig. S1). Consistent with our results, it has been found that decreasing the mRNA expression level of MAOB can extend the survival time of glioblastoma [44, 45]. In addition, MAOB can be used as a novel biomarker to predict the prognosis of colorectal carcinoma [46] and can affect the progression of esophageal cancer [47]. MAOB can also be used as a novel target for the treatment of prostate cancer [48] and presents differential expression in oral tumors [49]. In conclusion, MAOB may be regarded as a biomarker of BC. However, there are no reports about the role of MAOB in BC. Thus, more studies are needed to clarify the role of MAOB in the occurrence and development of BC.

FASN, a key biosynthetic enzyme involved in lipogenesis and the production of longchain fatty acids from acetylcoenzyme A and malonyl-CoA, plays a key role in energy metabolism [50]. It has been regarded as a potential target for the treatment of prostate cancer, thyroid cancer and multiple myeloma [51–53]. Moreover, the expression of FASN is involved in the progression of BC [54]. In particular, FASN has been suggested to be upregulated in BC and to be associated with the histologic grade and recurrence of BC [54, 55], which was consistent with our findings. Moreover, inhibition of FASN expression can suppress the migration capacity of bladder transitional cell carcinoma by activating AKT [56]. Thus, our study further highlights the role of FASN in the occurrence and development of BC.

LRP1, a ubiquitously expressed cell surface receptor, can regulate the lipoprotein metabolism and protease homeostasis [57]. Consistently, LRP1 has been related to the poor prognosis of clear-cell renal cell carcinoma [58]. In addition, LRP1 mutation plays a key role in the occurrence of gastric cancer [59]. It has been demonstrated that the tPA-LRP1 pathway is a key switch for regulating the progression of melanoma by affecting the cellular composition and proteolytic makeup of the tumor niche [60]. More importantly, the expression of LRP1 is involved in the outcome of lung adenocarcinoma [61]. Therefore, LRP1 may be a therapeutic target of BC.

Interestingly, we found that the expression levels of both MAOB and LRP1 were downregulated in BC compared with normal samples, while low expression was associated with prolonged OS (Fig. 7 and Fig. S1). Conversely, FASN was upregulated in BC compared with normal samples, while high expression of FASN was involved in poor OS (Fig. 7 and Fig. S1). Consistent with our research, previous study have revealed that CXCL11 expression is significantly upregulated in colon adenocarcinoma, and upregulation of CXCL11 expression is associated with a better prognosis [62]. Moreover, it was speculated that the paradox between the significance of expression and prognosis for CXCL11 might be due to the regulatory complexity (62). Here, we speculate that MAOB and LRP1 may not affect the occurrence of BC, but may be associated with the development of BC. In brief, BC occurrence may decrease the expression levels of MAOB and LRP1. Inversely, FASN may be related to the occurrence of BC. However, more research is needed.

### Conclusion

In conclusion, based on data from the TCGA and GEO databases, we developed a novel MRG signature to predict the survival of BC. Furthermore, a nomogram integrating the gene signature, age and tumor stage was constructed to preferably predict the survival of BC patients. Moreover, quantitative real-time PCR suggested that the expression levels of MAOB and LRP1 were downregulated in cancer tissues compared with paracarcinoma tissues, and the expression of FASN was upregulated. Therefore, these findings revealed that MAOB, LRP1 and FASN may play key roles in BC by regulating metabolism. However, more research is needed to illustrate their roles in BC.

## Supplementary Information

**Additional file 1: Figure S1.** The KM survival curves of MAOB, FASN and LRP1 in the training, testing and validation sets. (A) The training set. (B) The testing set (C) The validation set.

**Additional file 2: Table S1.** The primer sequences and annealing temperatures of MAOB, FASN and LRP1.

**Additional file 3: Table S2.** The metabolism-related processes and pathways enriched by h.all.v7.2.entrez.gmt gene set.

**Additional file 4: Table S3.** The DEGs between normal and cancer samples in GSE13507.

**Additional file 5: Table S4.** The DEGs between normal and cancer samples in TCGA database.

**Additional file 5: Table S5.** Twenty-seven metabolism-related DEGs.

**Additional file 7: Table S6.** The survival analysis between mutated and nonmutated samples of each mutated gene among the 27 metabolism-related DEGs.

## Data Availability

The GSE13507 dataset used in the present study can be found in GEO database (https://www.ncbi.nlm.nih.gov/geo/), and the dataset of TCGA (https://portal.gdc.cancer.gov/) is available.

## Abbreviations

BC: Bladder cancer
MRG: Metabolism-related gene
KM: Kaplan-Meier
ROC: Receiver operating characteristic
LRP1: LDL Receptor Related Protein 1
MAOB: Monoamine Oxidase B
FASN: Fatty Acid Synthase
FOXJ1: Forkhead Box J1
SAT1: Spermidine/Spermine N1-Acetyltransferase 1
ASS1: Argininosuccinate Synthase 1
GEO: Gene-Expression Omnibus
TCGA: The Cancer Genome Atlas
GSEA: Gene set enrichment analysis
DEGs: Differentially expressed genes
GO: Gene Ontology
BP: Biological process
MF: Molecular function
CC: Cellular component
KEGG: Kyoto Encyclopedia of Genes and Genomes
PPI: Protein-protein interaction
AUC: Area under curve
cDNA: Complementary DNA
GAPDH: Glyceraldehyde-3-Phosphate Dehydrogenase
TYMS: Thymidylate Synthetase
TK1: Thymidine Kinase 1
DPYD: Dihydropyrimidine Dehydrogenase
UCK2: Uridine-Cytidine Kinase 2
AKT: AKT Serine/Threonine Kinase
