# Supplementary figures and images for: Identification of a novel metabolism-related gene signature associated with the survival of bladder cancer
Source: BMC Cancer. 2021 Nov 24;21:1267. doi: 10.1186/s12885-021-09006-w (PMC8611960; doi:10.1186/s12885-021-09006-w)

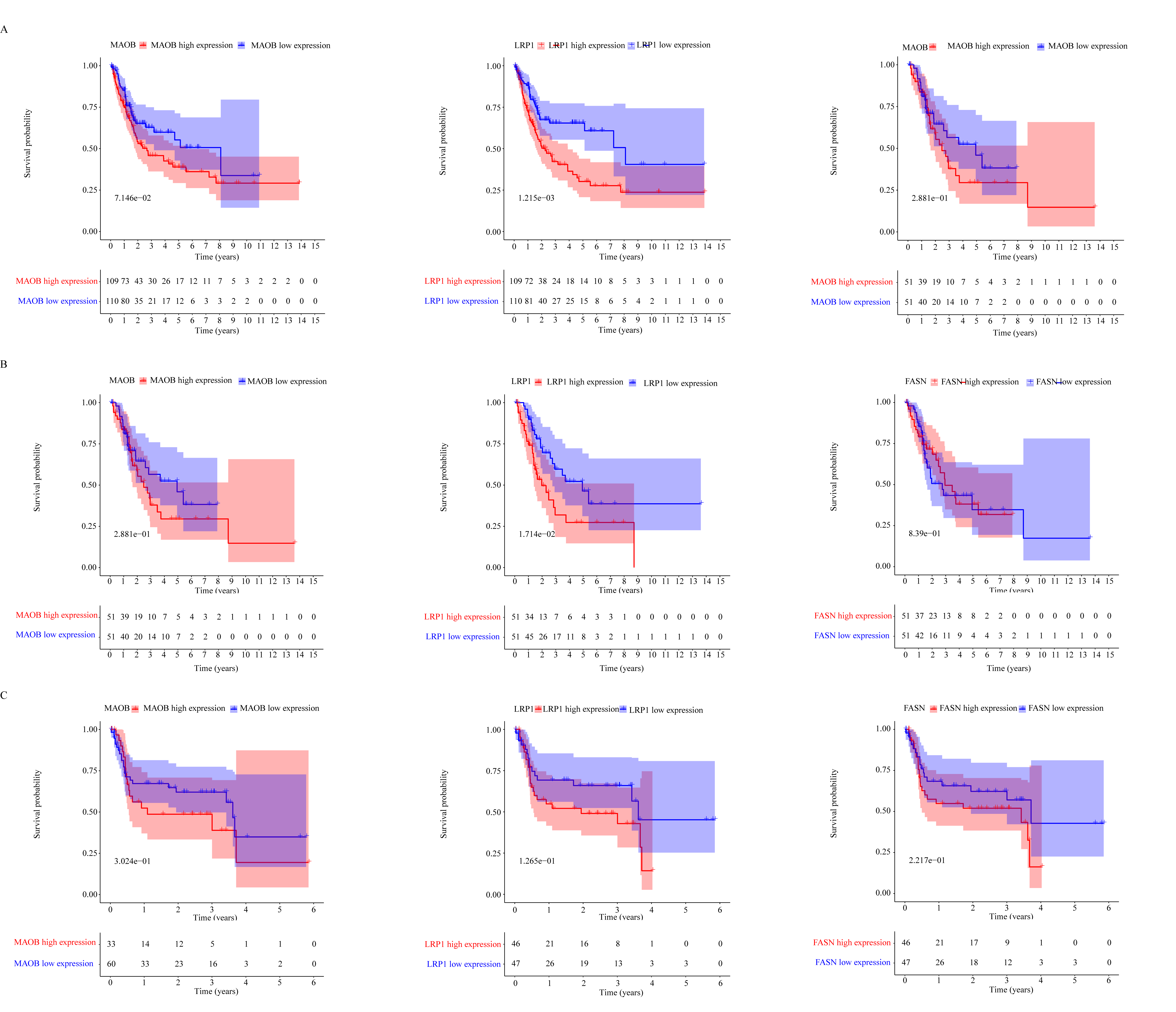

Supplement: Supplementary file 1 — Additional file 1: Figure S1. The KM survival curves of MAOB, FASN and LRP1 in the training, testing and validation sets. (A) The training set. (B) The testing set (C) The validation set. [file 12885_2021_9006_MOESM1_ESM.tif]
