# Supplementary material for: Identification of a novel metabolism-related gene signature associated with the survival of bladder cancer
Source: BMC Cancer. 2021 Nov 24;21:1267. doi: 10.1186/s12885-021-09006-w (PMC8611960; doi:10.1186/s12885-021-09006-w)
Supplement: Supplementary file 2 — Additional file 2: Table S1. The primer sequences and annealing temperatures of MAOB, FASN and LRP1. [file 12885_2021_9006_MOESM2_ESM.docx]

Table S1 The primer sequences and annealing temperatures of MAOB, FASN and LRP1

| Gene | Sequence（5^，^→3^,^） | | Length | Tm | GC% |
| --- | --- | --- | --- | --- | --- |
| MAOB | Forward Primer | CAATCCCCCTCTGCCAATGA | 20 | 55.64 | 55 |
|  | Reverse Primer | TCCAACGTGTAGGCAACTGG | 20 | 57.13 | 55 |
| FASN | Forward Primer | GCAAGCTGAAGGACCTGTCT | 20 | 59.96 | 55 |
|  | Reverse Primer | AATCTGGGTTGATGCCTCCG | 20 | 60.11 | 55 |
| LRP1 | Forward Primer | TCTGCTTCGTGTGCCTATCC | 20 | 59.82 | 55 |
|  | Reverse Primer | CAGTCATTGTCATTGTCGCATCT | 23 | 59.63 | 43.48 |
